# Aberrant methylation of Serpine1 mediates lung injury in neonatal mice prenatally exposed to intrauterine inflammation

**DOI:** 10.1186/s13578-022-00901-8

**Published:** 2022-10-01

**Authors:** Dongting Yao, Jiuru Zhao, Qianqian Zhang, Tao Wang, Meng Ni, Sudong Qi, Qianwen Shen, Wei Li, Baihe Li, Xiya Ding, Zhiwei Liu

**Affiliations:** 1 Departments of Neonatology, International Peace Maternity and Child Health Hospital of China Welfare Institution, School of Medicine, Shanghai Jiao Tong University, 910# Hengshan Road, Shanghai, 20030 China; 2 Shanghai Key Laboratory of Embryo Original Diseases, Shanghai Jiao Tong University, Shanghai, China; 3 Department of Laboratory Medicine, Longhua Hospital, Shanghai University of Traditional Chinese Medicine, Shanghai, China

**Keywords:** Serpine1, Intrauterine inflammation, Lung injury, Cell senescence, Hypomethylation

## Abstract

**Background:**

Intrauterine inflammation (IUI) alters epigenetic modifications in offspring, leading to lung injury. However, the epigenetic mechanism underlying IUI-induced lung injury remains uncertain. In the present study, we aim to investigate the effect of IUI on lung development, and to identify the key molecule involved in this process and its epigenetic regulatory mechanism.

**Results:**

Serpine1 was upregulated in the lung tissue of neonatal mice with IUI. Intranasal delivery of *Serpine1* siRNA markedly reversed IUI-induced lung injury. Serpine1 overexpression substantially promoted cell senescence of both human and murine lung epithelial cells, reflected by decreased cell proliferation and increased senescence-associated β-galactosidase activity, G0/G1 cell fraction, senescence marker, and oxidative and DNA damage marker expression. IUI decreased the methylation level of the *Serpine1* promoter, and methylation of the promoter led to transcriptional repression of *Serpine1*. Furthermore, IUI promoted the expression of Tet1 potentially through TNF-α, while Tet1 facilitated the demethylation of *Serpine1* promoter. DNA pull-down and ChIP assays revealed that the *Serpine1* promoter was regulated by Rela and Hdac2. DNA demethylation increased the recruitment of Rela to the *Serpine1* promoter and induced the release of Hdac2.

**Conclusion:**

Increased Serpine1 expression mediated by DNA demethylation causes lung injury in neonatal mice with IUI. Therefore, therapeutic interventions targeting Serpine1 may effectively prevent IUI-induced lung injury.

**Supplementary Information:**

The online version contains supplementary material available at 10.1186/s13578-022-00901-8.

## Background

Intrauterine inflammation (IUI), a severe inflammatory complication of pregnancy, is one of the leading causes of preterm birth (PTB) [1] and contributes to 25–40% of PTBs, with an incidence of more than 90% in extremely preterm infants [2]. PTB is the major cause of death in children younger than five years worldwide [3], largely owing to impaired respiration [4]. Bronchopulmonary dysplasia (BPD), the most common complication of PTB, presents with lung injury with defective gas exchange resulting from alveolar simplification and vascular dysregulation [5]. In pregnancies with IUI, exposure to a high level of inflammatory factors, including tumor necrosis factor (TNF)-α and interleukin (IL)-6, affects the normal course of fetal lung development, causing damage to immature lung tissue and disorder of post-injury repair [6]. However, the mechanisms underlying IUI-induced lung injury remain largely unknown.

The “developmental origins of the health and disease” hypothesis suggest that an adverse environment in utero can increase the risk of adult disease through fetal reprogramming [7]. Environmental exposures in early life (during pregnancy and the newborn period), such as nutritional deficiency and pollutants, reprogram gene expression primarily through epigenetic modifications. Thus, IUI may affect lung development by altering epigenetic modifications, which include DNA methylation, histone modifications, and non-coding RNAs. DNA methylation is widely involved in transcriptional regulation during development and differentiation [8]. It is catalyzed by the methyltransferases DNMT3a/b and maintained by DNMT1 during DNA replication, while demethylation is catalyzed by the dioxygenase ten-eleven translocation family enzyme TET1-3 [9]. Studies have confirmed that DNA methylation is closely related to lung development and affected by environmental stimuli [10, 11].

Our pilot study showed a significant upregulation of serine or cysteine peptidase inhibitor clade E member 1 (*Serpine1*) in the lung tissue of neonatal mice with IUI, suggesting that *Serpine1* may be a candidate for mediating IUI-induced lung injury. Serpine1, also known as plasminogen activator inhibitor-1, is a major inhibitor of tissue plasminogen activator and urokinase [12], and is involved in hemostasis, extracellular proteolysis, cell migration, and tissue remodeling. *Serpine1* is highly conserved in humans and mice [13], and its epigenetic modification status is associated with the development of cardiovascular disease, diabetes, ageing, tissue fibrosis, and inflammation [14–18]. In pulmonary fibrosis, studies indicated that Serpine1 promoted the proliferation of fibroblasts and senescence of type 2 alveolar epithelial cells through the p53-p21 signaling pathway [19, 20]. However, the role of *Serpine1* in lung development remains unidentified.

This study investigates the effects of IUI on lung development in neonatal mice and identify the key molecule and the underlying epigenetic regulatory mechanisms. The results will provide insights into the molecular mechanism of IUI leading to progeny BPD and suggest potential targets for disease prevention and control.

## Results

### IUI impaired lung development

**Fig. 1:**
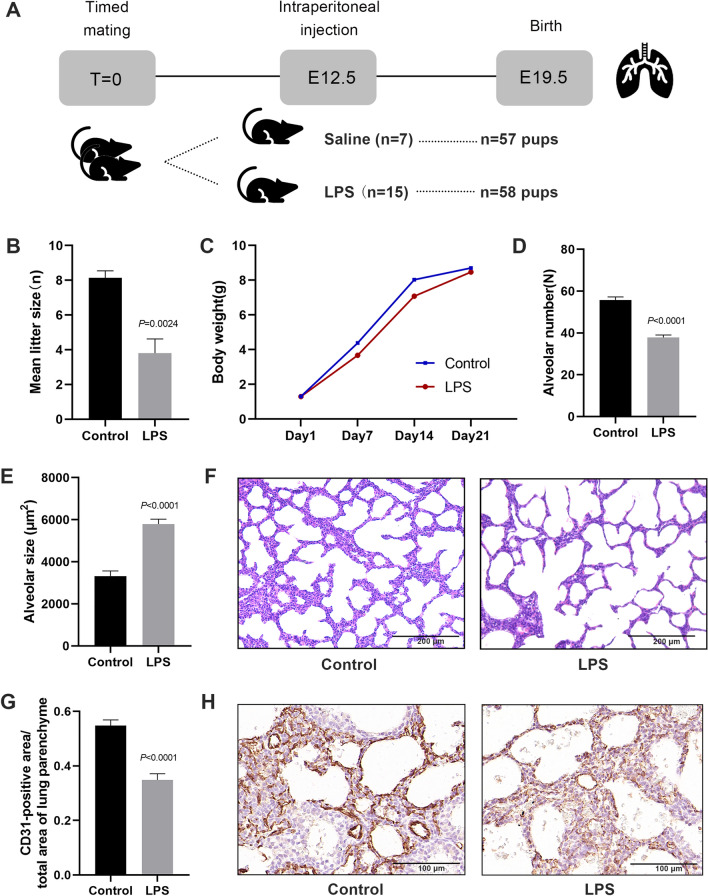
IUI impaired lung development. **A** Pregnant C57BL/6 mice were intraperitoneally injected with either LPS or saline on E12.5. **B**, **C** Mortality and mean body weight of neonatal mice. **D–F** Measurement of alveolar number and alveolar size in the lung tissues of neonatal mice. Representative image of the lung tissue stained with hematoxylin and eosin. Scale bars, 200 μm. **G, H** Immunohistochemistry detection of CD31 in the lung tissues of neonatal mice. Scale bars, 100 μm. Data show mean ± SEM; Data was analysed using unpaired *t*-tests

An effective IUI model was established by injecting lipopolysaccharide (LPS) into 15 dams on E12.5, with 58 litters surviving, and 7 dams with sterile normal saline, with 57 litters surviving (Fig. 1A). The average litter size per dam was 3.86 and 8.14 pups in the LPS and control groups, respectively (Fig. 1B). Pups in the LPS group showed a decrease in the body weight compared to those in the control group between P1 and P14, with no statistical difference (Fig. 1C). Histological examination of the lung tissues demonstrated fewer alveoli and an enlargement of the alveolar space in neonatal mice with IUI compared to those in the control group mice (Fig. 1D–F). A semi-quantitative analysis further revealed a 0.68-fold decrease in alveolar number induced by IUI (Fig. 1D) and an associated 1.72-fold increase in alveolar size (Fig. 1E). Immunohistochemistry for CD31 showed a decreased vascular density with a 0.63-fold decrease in CD31-positive area in neonatal mice with IUI compared with the control (Fig. 1G, H). Studies have shown that IUI can enhance surfactant protein and lipid synthesis, thereby promoting lung maturation [21, 22]. Consistently, the immunofluorescence assay of Pro-SFTPC showed that more positive cells were present in the lung tissue of neonatal mice with IUI than in that of controls (Additional file 1: Fig. S1A, B). These results indicate that IUI impaired the normal process of lung development.

### Serpine1 mediated lung injury in IUI mice

To identify the key molecule that mediates IUI-induced lung injury, RNA sequencing of lung tissues was performed. The results revealed that 283 and 149 genes were upregulated and downregulated in neonatal mice with IUI, respectively (*Q*-value ≤ 0.05 and fold-change ≥ 2). Gene ontology (GO) term enrichment analysis showed that the upregulated expressed genes were mainly involved in positive regulation of leukocyte chemotaxis, chemokine-mediated, chemokine activity and positive regulation of inflammatory response (Additional file 1: Fig. S1C). Kyoto Encyclopedia of Genes and Genomes (KEGG) pathway enrichment analysis revealed that the upregulated expressed genes were enriched in IL-17, TNF, toll-like receptor, and other signaling pathways (Additional file 1: Fig. S1D) involved in inflammatory response and autoimmunity [23]. GO and KEGG analyses of the downregulated expressed genes showed that they were mainly enriched in muscle contraction, PPAR signaling and inflammatory mediator regulation of TRP channels (Additional file 1: Fig. S1E, F). These results indicated that the maternal LPS exposure induced an inflammatory response and effected the cell development in the lung of neonatal mice.

**Fig. 2:**
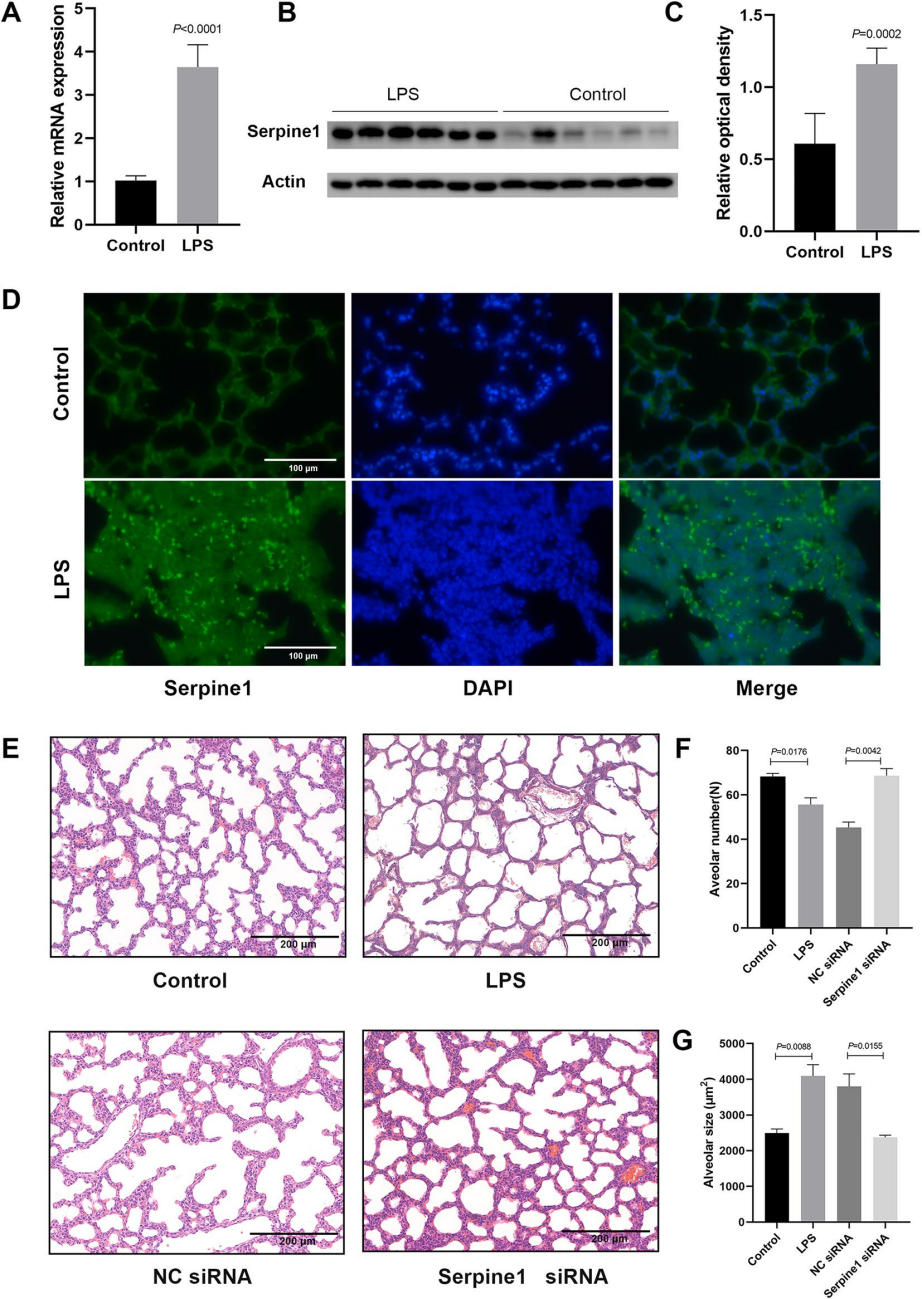
Intranasal delivery of *Serpine1* siRNA reversed lung injury induced by IUI. **A** The mRNA expression of *Serpine1* analysed using RT-qPCR in lung tissues of neonatal mice. **B**,** C** Serpine1 expression analysed using western blot in lung tissues of neonatal mice. **C** Immunofluorescence detection of Serpine1 in lung tissues of neonatal mice. (green, DAPI: blue). Scale bars, 100 μm. **E–G** Histological examination of siRNA-treated neonatal mouse lungs. The lung tissue was stained with hematoxylin and eosin. The alveolar number and alveolar size were quantified. Scale bars, 200 μm. Data show mean ± SEM; Data was analysed using unpaired *t*-tests

RT-qPCR analysis of the top differentially expressed genes validated the upregulation of *Serpine1*, nuclear receptor subfamily 4 group A member 1 (*Nr4a1*), FBJ osteosarcoma oncogene B (*FosB*), early growth response 1 (*Egr1*), and protein phosphatase 1 regulatory inhibitor subunit 3B (*Ppp1r3b*), as well as the downregulation of death-associated protein kinase 2 (*Dpcr1*) and gastrin-releasing peptide receptor (*Grpr*) (Fig. 2A, Additional file 2: Fig. S2A). Western blot analysis showed significant upregulation of Serpine1 and Nr4a1 expression in the lung of neonatal mice with IUI (Fig. 2B, C, Additional file 2: Fig. S2B, C). Besides, *Serpine1* showed much more increased expression than *Nr4a1*. Therefore, we focused on *Serpine1* in the subsequent experiments. The postnatal expression kinetics of *Serpine1* were further examined. Results showed that *Serpine1* hyperexpression persisted for at least 21 days after birth (Additional file 2: Fig. S2D). Immunofluorescence verified the hyperexpression of *Serpine1* in the lung of neonatal mice with IUI. Meanwhile, Serpine1 was mainly distributed in lung epithelial cells (Fig. 2D).

To further verify the role of *Serpine1* in lung injury in neonatal mice with IUI, an siRNA approach was used to inhibit *Serpine1* function in vivo. *Serpine1* siRNA was intranasally delivered for four consecutive days from P3 to P6 [24–27], and mice were euthanized four days after gene therapy (one week after birth). As shown in Additional file 2: Fig. S2E–G, Serpine1 expression was significantly decreased in the lungs treated with *Serpine1* siRNA compared with that in the scrambled siRNA group at both mRNA and protein levels. As expected, histological examination showed that the inhibition of *Serpine1* reversed the impaired alveolarization, indicated by the decreased alveolar size and increased alveolar count (Fig. 2E–G) in the lungs of neonatal mice with IUI compared with those in the scrambled siRNA group. These results suggest that Serpine1 may be a potential therapeutic target for IUI-induced alveolar injury.

### *Serpine1* promoted senescence of lung epithelial cells

**Fig. 3:**
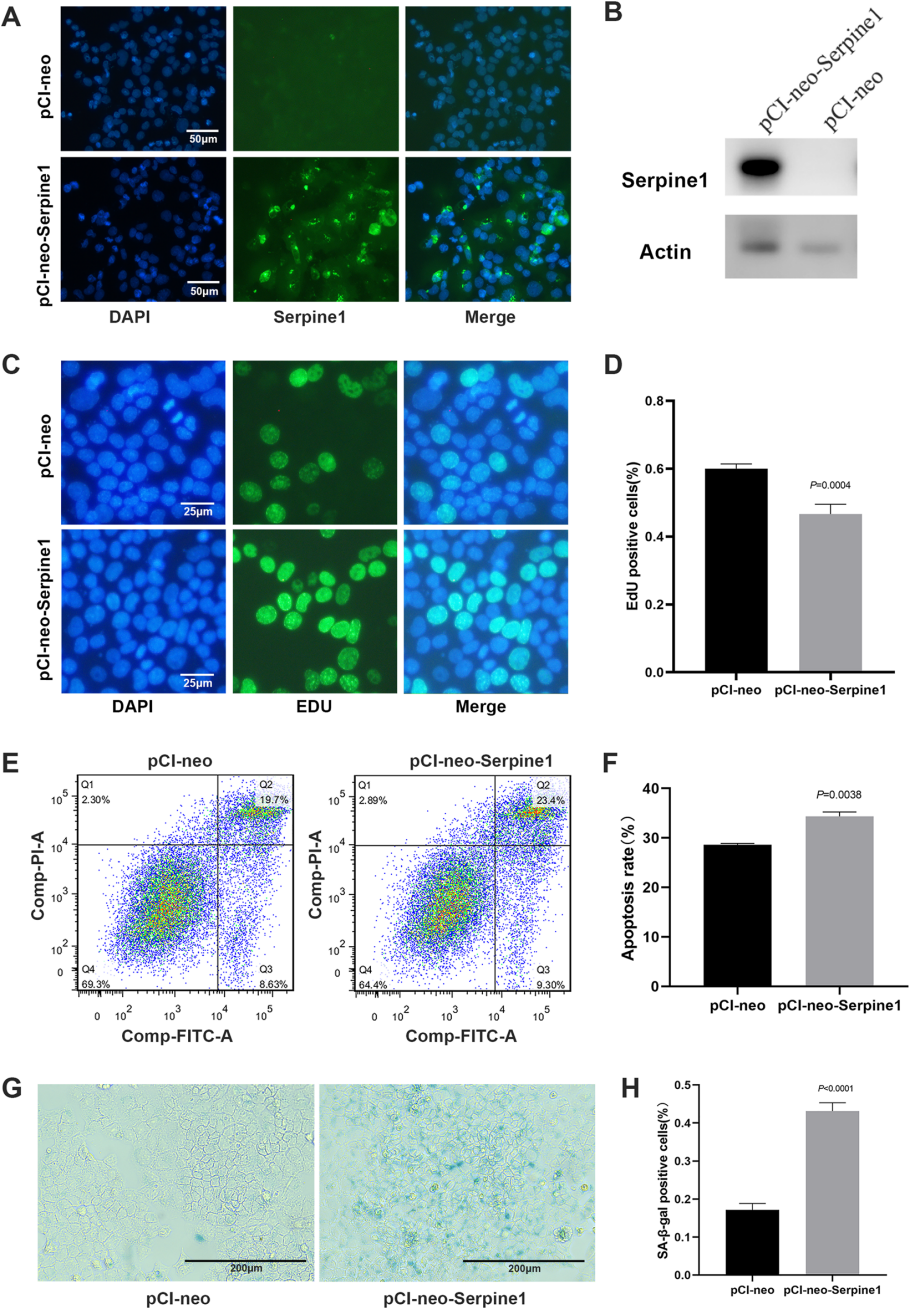
Serpine1 decreased the proliferation of MLE-12 cells. **A**, **B** *Serpine1* expression analysed using immunofluorescence and western blot in *Serpine1*-overexpressing MLE-12 cells. Scale bars, 50 μm. **C**, **D** Proliferation analysed using the EdU assay (green, DAPI: blue) in Serpine1-overexpressing MLE-12 cells. Scale bars, 25 μm. **E**,** F** Apoptosis rate analysed using flow cytometry in Serpine1-overexpressing MLE-12 cells. **G**,** H** SA-b-gal activity measured through X-gal in Serpine1-overexpressing MLE-12 cells. Scale bars, 200 μm. Data show mean ± SEM; Data was analysed using unpaired *t*-tests

**Fig. 4:**
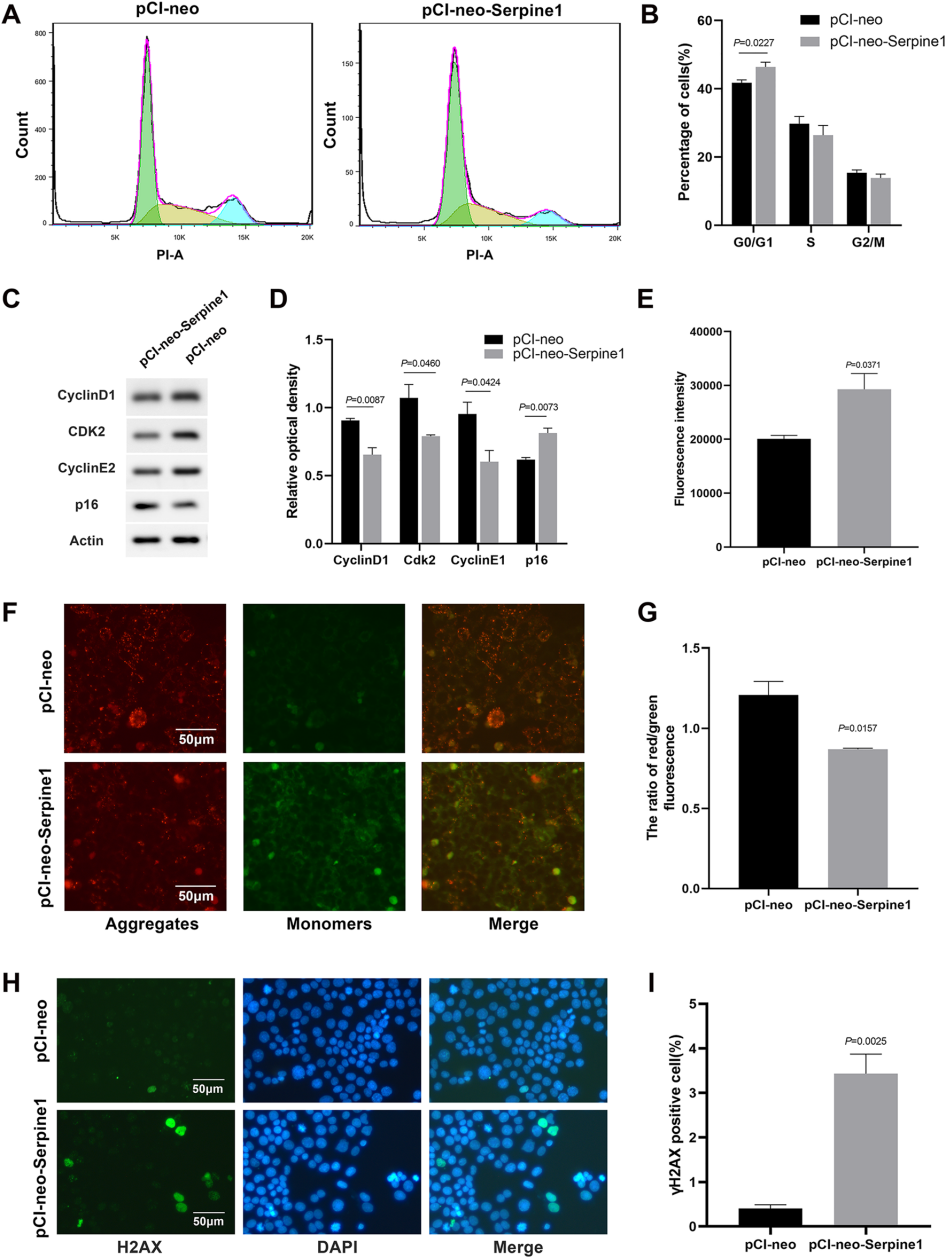
Serpine1 promoted the senescence of MLE-12 cells. **A**,** B** Cell cycle of *Serpine1*-overexpressing MLE-12 cells analysed using flow cytometry. Cell fractions in the G0/G1, G2/M, and S phases were calculated. **C**,** D** CCND1, CDK2, CCNE2, and p16 expressions analysed using western blot in *Serpine1*-overexpressing MLE-12 cells. **E** The intracellular ROS content in *Serpine1*-overexpressing MLE-12 cells measured using the fluorescent probe DCFDA. **F**,** G** JC-1 staining in *Serpine1*-overexpressing MLE-12 cells. Scale bars, 50 μm. The JC-1 red/green signal ratio was calculated using a fluorescence plate reader. **H**,** I** γH2AX expression in *Serpine1*-overexpressing MLE-12 cells determined using immunofluorescence (green, DAPI: blue). Scale bars, 50 μm. Data show mean ± SEM; Data was analysed using unpaired *t*-tests

To determine the function of Serpine1 in alveolar cells, the *Serpine1* overexpression plasmid pCI-neo-Serpine1 was constructed and transfected into MLE-12 cells (positive cells), an alveolar epithelial cell line (Fig. 3A, B, Additional file 3: Fig. S3A). Transcriptome sequencing showed that *Serpine1* regulated several key genes enriched in cellular senescence (Additional file 4: Fig. S4A–D). Results showed that *Serpine1* overexpression significantly decreased the percentage of EdU-positive MLE-12 cells (60% VS 47%, Fig. 3C, D), increased the apoptosis rate (28.6 VS 34.4%, Fig. 3E, F) and senescence-associated β-galactosidase activity in positive cells (17 VS 43%, Fig. 3G, H). These results indicated that Serpine1 promoted the senescence of MLE-12 cells. Cell senescence would arrest the cell cycle and inhibit cell proliferation [28]. Flow cytometric analysis showed that positive cells had an increased G0/G1 phase cell fraction (Fig. 4A, B). Western blot further demonstrated Serpine1 repressed the expression of cell cycle-related proteins cyclin D1 (CCND1), cyclin E2 (CCNE2), and cyclin-dependent kinase 2 (CDK2), while increased the expression of senescence marker p16 (Fig. 4C, D). Cell senescence is often induced by dysregulation of reactive oxygen species (ROS) production, mitochondrial membrane potential, and DNA fragmentation. Results showed that Serpine1 increased ROS generation as measured by DCFDA staining (Fig. 4E), decreased JC-1 aggregates (red), and increased JC-1 monomers (green) compared with the control vector-treated group (Fig. 4F, G). The magnitude of DNA damage after transfection was further analysed via γH2AX immunostaining (Fig. 4H). The percentage of MLE-12 cells with γH2AX accumulation increased from < 0.5% in the controls to 3.4% in positive cells (Fig. 4I).

**Fig. 5:**
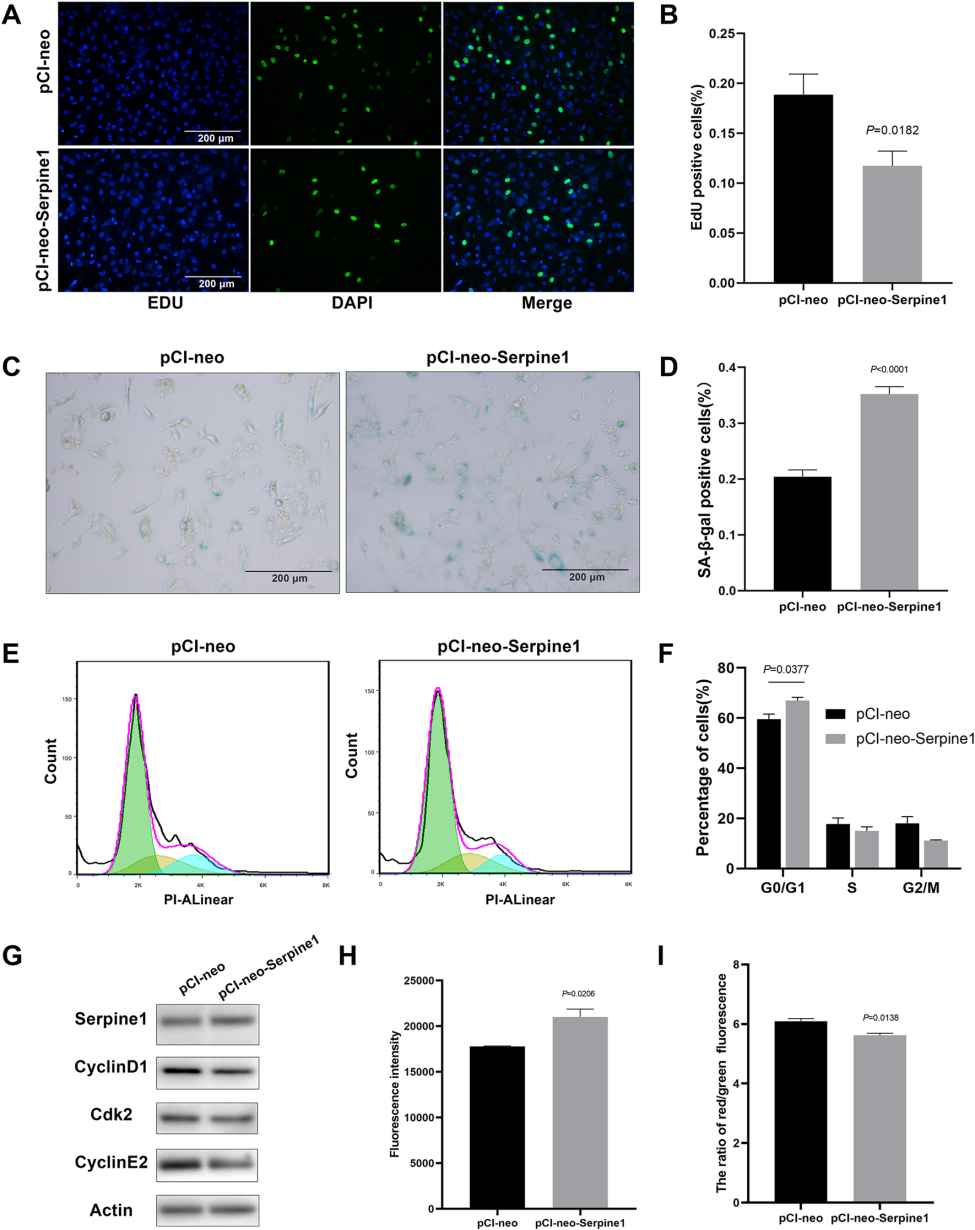
Serpine1 promoted the senescence of A549 cells. **A**,** B** Proliferation analysed using the EdU assay (green, DAPI: blue) in Serpine1-overexpressing A549 cells. Scale bars, 200 μm. **C**,** D** SA-b-gal activity measured through X-gal in Serpine1-overexpressing A549 cells. Scale bars, 200 μm. **E**,** F** Cell cycle of *Serpine1*-overexpressing A549 cells analysed using flow cytometry. Cell fractions in the G0/G1, G2/M, and S phases were calculated. **G** Seripine1, CCND1, CDK2, and CCNE2 expressions analysed using western blot. **H** The intracellular ROS content in *Serpine1*-overexpressing A549 cells measured using the fluorescent probe DCFDA. **I** JC-1 staining in *Serpine1*-overexpressing A549 cells. The JC-1 red/green signal ratio was calculated using a fluorescence plate reader. Data show mean ± SEM; Data was analysed using unpaired *t*-tests

To further ascertain the function of Serpine1 in human lung epithelial cells, we investigate the effect of *Serpine1* overexpression in A549 cells, a pulmonary epithelial cell line derived from a human alveolar cell carcinoma. Consistently, the results showed that *Serpine1* overexpression (Additional file 3: Fig. S3B–D) inhibited the proliferation (Fig. 5A, B), promoted the senescence of A549 cells (Fig. 5C, D), arrest the cell cycle (Fig. 5E–G), increased ROS generation (Fig. 5H), decreased JC-1 aggregates (red), and increased JC-1 monomers (green) (Fig. 5I), but did not significantly affect cellular apoptosis (Additional file 3: Fig. S3E, F) compared with the control vector-treated group. Nonetheless, the expression of CCND1, CCNE2 and CDK2 were all decreased (Fig. 5G). Besides, A549 is a p16-negative cell [29]. Thus, we detected the effect of *Serpine1* overexpression in BEAS-2B cells, a cell line derived from normal human bronchial epithelium. The result showed that *Serpine1* increased the expression of senescence marker p16 (Additional file 3: Fig. S3G). Collectively, these results suggested that cellular senescence induced by *Serpine1* might be responsible for the epithelial injury after exposure to IUI.

### Tet1 induced hypomethylation of *Serpine1* promoter

**Fig. 6:**
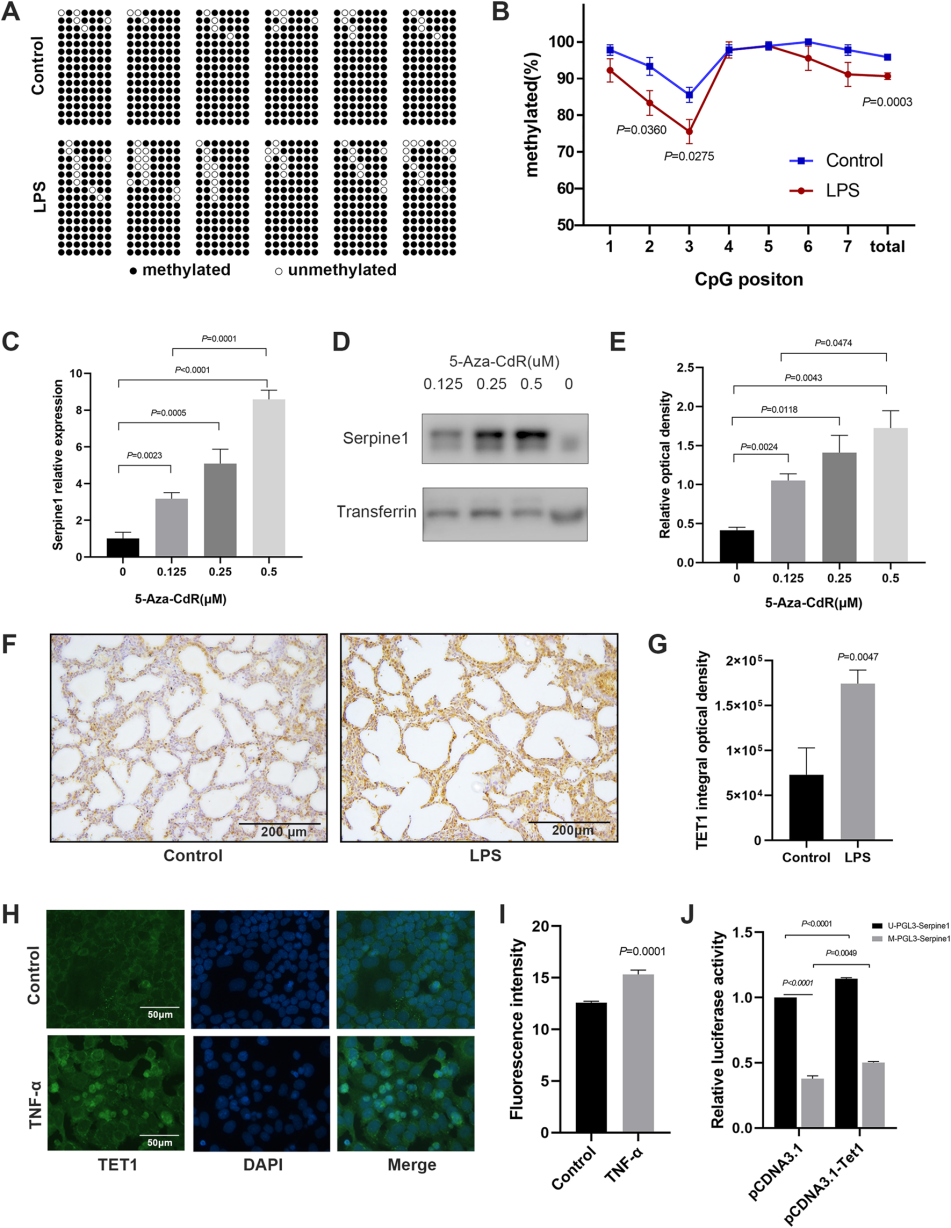
*Serpine1* expression was regulated by TNF-α-Tet1 pathway. **A**,** B** DNA methylation levels analysed on the CpG island of the *Serpine1* promoter using bisulfite sequencing PCR in lung tissues of neonatal mice. **C-E** *Serpine1* expression analysed using RT-qPCR and western blot in 5-Aza-CdR-treated MLE-12 cells. **F, G** Tet1 expression in lung tissues of neonatal mouse determined using immunohistochemistry. Scale bars, 200 μm. **H**,** I** Tet1 expression in TNF-α-treated MLE-12 cells determined using immunofluorescence (green, DAPI: blue). Scale bars, 50 μm. **J** The effects of Tet1 on the transcriptional efficiency of the Serpine1 promoter using luciferase assay. Data show mean ± SEM; Data was analysed using unpaired *t*-tests and one-way ANOVA

Bioinformatics analysis identified one CpG island in the *Serpine1* promoter. The bisulphite sequencing PCR further showed there was site-specific DNA methylation in the *Serpine1* promoter. The methylation of the *Serpine1* promoter reduced from 95.85 to 90.63% in neonatal mice with IUI compared to that in the control mice (Fig. 6A, B). To study the effect of DNA methylation on *Serpine1* expression, MLE-12 cells were treated with 5-Aza-2’-deoxycytidine (5-Aza-CdR), a DNA methyltransferase inhibitor. RT-qPCR and western blot demonstrated that 5-Aza-CdR promoted *Serpine1* expression in a dose-dependent manner (Fig. 6C–E). Next, to identify the related enzymes modulating *Serpine1* DNA methylation, RT-qPCR and immunohistochemistry analyses were performed in lung tissues. Results showed that the expression of Tet1 was significantly increased in neonatal mice with IUI, but not Dnmt1, Dnmt3a, Mbd1 (Fig. 6F–G, Additional file 5: Fig. S5A). Reports demonstrated that TNF-α can promote *Serpine1* expression in endothelial cells [30]. In this study, we found that IUI induced the upregulation of TNF-α in the maternal serum(from 25.54 ± 1.731 pg/ml to 97.22 ± 12.19 pg/ml, *P* = 0.0043), placental tissue (from 1298 ± 24.82 pg/ml to 1405 ± 23.40 pg/ml, *P* = 0.0201), and neonatal mouse lung tissue (1.33-fold, Additional file 5: Fig. S5B). Thus, the effect of TNF-α was examined in MLE-12 cells. The results showed that TNF-α increased the expression of Tet1 (Fig. 6H, I, Additional file 5: Fig. S5C) and Serpine1 (Additional file 5: Fig. S5D). To determine whether Tet1 affect the methylation of the *Serpine1* promoter, vector constructs harboring the *Serpine1* promoter region were methylated in vitro using the CpG methyltransferase *M.SssI*. Results showed that methylation of the *Serpine1* promoter caused a remarkable decrease in the luciferase activity, while Tet1 rescued the activity (Fig. 6J). Therefore, TNF-α might promote Serpine1 expression through Tet1 upregulation.

### Methylation of *Serpine1* promoter prevented transcriptional activation

**Fig. 7:**
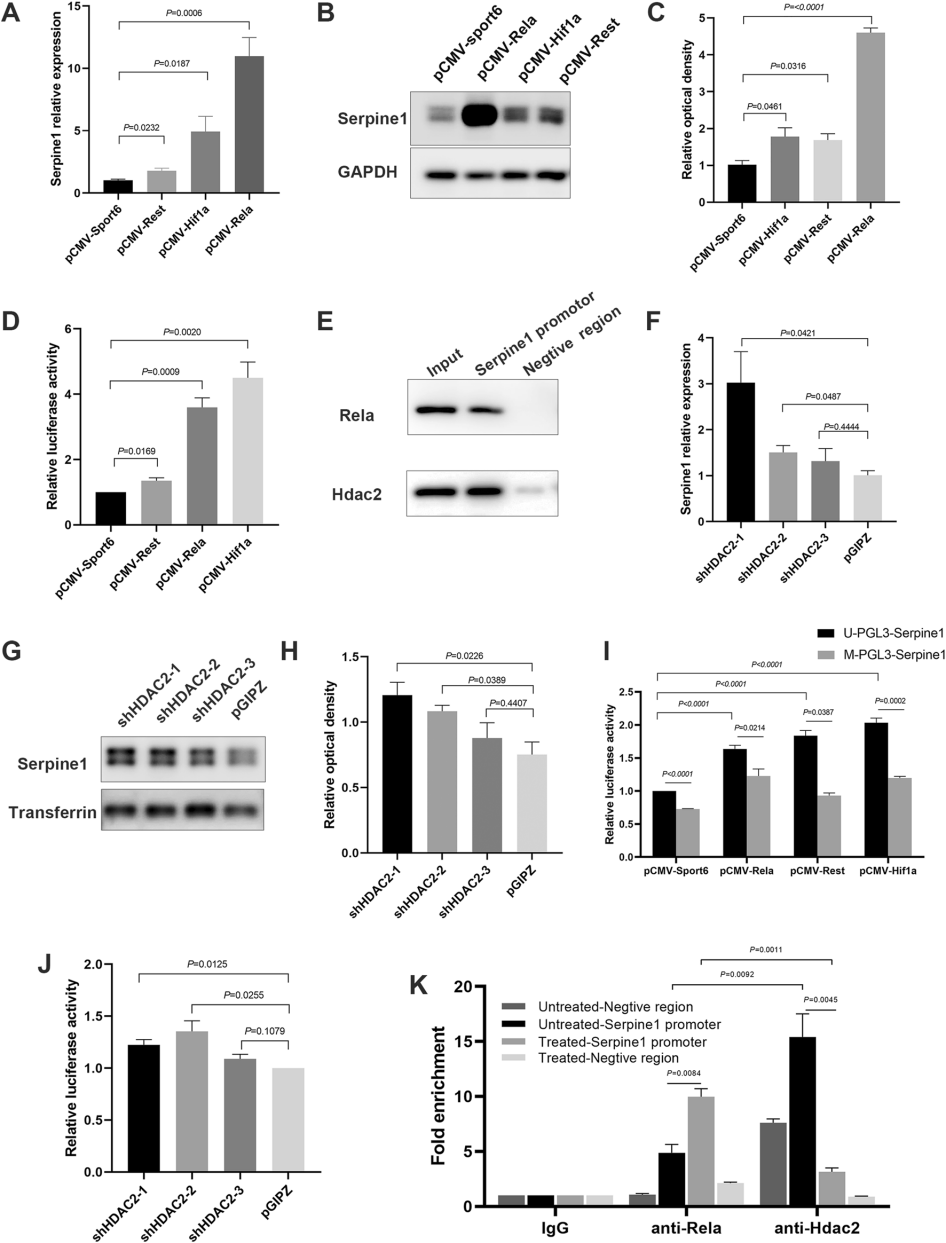
Demethylation promoted Rela but inhibited Hdac2 binding to the *Serpine1* promoter. **A–C** *Serpine1* expression analysed using RT-qPCR and western blot in Rela-, Hif1a-, and Rest-overexpressing MLE-12 cells. **D** Transcriptional activity of Rela, Hif1a, and Rest at the *Serpine1* promoter analysed using luciferase assay in MLE-12 cells. **E** The binding ability of Rela and Hdac2 to the biotin-labelled *Serpine1* promoter probe analysed via DNA pull-down assay. **F–H** Serpine1 expression analysed using RT-qPCR and western blot in Hdac2-knockdown MLE-12 cells. **I** Transcriptional activity of Rela, Hif1a, and Rest at the methylated-Serpine1 promoter analysed using luciferase assay in HEK293T cells. **J** Transcriptional activity of Rela at the *Serpine1* promoter analysed using luciferase assay in Hdac2-knockdown MLE-12 cells. **K** The enrichment of TFs at the *Serpine1* promoter region determined using anti-Rela and anti-Hdac2 antibodies via ChIP-qPCR in 5-Aza-CdR-treated MLE-12 cells. Data show mean ± SEM; Data was analysed using unpaired *t*-tests

To explore the potential mechanism of DNA methylation on *Serpine1* expression, transcription factor (TF) binding at the *Serpine1* promoter was firstly screened using bioinformatics. Fifteen potential TFs were then identified. A luciferase assay was used to verify the binding of predicted TFs to the *Serpine1* promoter. The results showed that Rest, Hif1a, and Rela could activate the transcription of the *Serpine1* promoter in MLE-12 cells (Fig. 7A–D). Next, a DNA affinity pull-down assay was performed to identify the protein bind to *Serpine1* promoter using a biotinylated probe encompassing the −2000 to −1 bp promoter region. Western blot of the precipitated proteins showed that Rela did bind to the *Serpine1* promoter (Fig. 7E, upper). Interestingly, we identified another protein, histone deacetylase 2 (Hdac2), through LC-MS (Additional file 6: Fig. S6A, B) and western blot (Fig. 7E, lower), that directly interacted with the *Serpine1* promoter. Hdac2 is a histone deacetylase enzyme, which removes the acetyl group from histones, and plays an important role in gene regulation [31]. To evaluate the effect of Hdac2 on *Serpine1* expression, MLE-12 cells were treated with trichostatin A (TSA), an Hdac2 inhibitor. The results showed that Serpine1 expression was significantly increased upon TSA treatment (Additional file 6: Fig. S6C–E). Besides, Hdac2 silencing by shRNA also increased *Serpine1* expression (Fig. 7F–H). These results suggested that *Serpine1* expression were regulated by Rela and Hdac2.

To determine whether DNA methylation repressed the transcriptional activity of TFs, vector constructs harboring the *Serpine1* promoter region were methylated in vitro using the CpG methyltransferase *M.SssI*. Results showed that methylation of the *Serpine1* promoter caused a moderate decrease in the transcriptional activity of Rest, Hif1a, and Rela (Fig. 7I). Further luciferase assay showed that Hdac2 silencing caused a significant increase in transcriptional activity of Rela on the *Serpine1* promoter (Fig. 7J). To investigate the interaction of Rela, Hdac2, and methylation of the *Serpine1* promoter, chromatin immunoprecipitation (ChIP) was performed in MLE-12 cells using corresponding ChIP-grade antibodies (Fig. 7K). Both Rela and Hdac2 were enriched in the *Serpine1* promoter, with Hdac2 showed a higher enrichment. After 5-Aza-CdR treatment, increased binding of Rela to the *Serpine1* promoter region was observed, whereas Hdac2 accumulation on the promoter was dramatically decreased. The results suggested that DNA demethylation caused Hdac2 to be removed from the *Serpine1* promoter, while Rela was recruited to it.

## Discussion

BPD is a chronic lung disease affecting the life and health of preterm infants [32]. Previous studies have demonstrated an association of BPD with adverse respiratory outcomes and neurodevelopmental dysfunctions in children [33, 34]. Moreover, clinical data have shown that IUI is associated with an increased risk of BPD at prematurity. Previous findings and our study suggest a possible role for IUI in inducing fetal and infant lung injury. BPD occurs owing to abnormal repair responses to prenatal and postnatal lung injury [35]. In this study, hematoxylin and eosin staining revealed abnormal lung morphology in neonatal mice with IUI, including fewer alveoli and simple alveolarization. Immunohistochemistry assay showed poor vascularization in neonatal mice with IUI. These pathological changes are similar to those observed in premature infants with BPD. In mice, lung development can be divided into five stages: embryonic (E9.5–12.5), pseudoglandular (E12.5–16.5), canalicular (E16.5–17.5), saccular (E17.5–PND5), and alveolar (PND5-28). In this study, E12.5 (the pseudoglandular period), marked by the formation of major duct systems, were selected to establish IUI, as it is prone to be influenced by multiple environmental factors. Intraperitoneal injection of LPS in pregnant C57BL/6 mice caused a higher mortality in neonatal mice, with significant changes in the signaling pathways regulating cellular inflammation, suggesting the successful establishment of an animal IUI model that can effectively delineate the relationship between IUI and BPD.

Recent studies demonstrated that cellular senescence in the alveolar epithelium contributes to the pathogenesis and progression of BPD [36, 37]. This study identified a significant upregulation of *Serpine1* in the lung tissue of neonatal mice with IUI. *Serpine1* overexpression promoted the senescence of both murine and human lung epithelial cells. Moreover, increased intracellular ROS generation and DNA damage induced by *Serpine1* overexpression confirmed its detrimental effect on cell survival. Accordingly, intranasal delivery of *Serpine1* siRNA markedly reversed IUI-induced lung injury. These results suggest that cellular senescence induced by *Serpine1* may be responsible for lung injury following exposure to IUI. Cellular senescence can be divided into stress-induced premature senescence (SIPS) and replicative senescence (RS). The p53-p21-pRB and p16-pRB pathways are responsible for transmitting cellular senescence signals [38], representing RS and SIPS, respectively [39]. We identified an association between *Serpine1*-induced senescence of alveolar epithelial cells and the accumulation of p16, indicating that *Serpine1* functioned through the SIPS pathway [40]. p21 and p16 belong to two cyclin-dependent kinase inhibitor (CKI) families. p16 inhibits the formation of CCND-CDK complexes and prevents cell cycle progression, while p53 activates p21 and inhibits CDK4/CCND and CDK2/CCNE complexes [41]. However, due to the decreased expression of CCNE2 in our study and the possible interaction between the two CKI families, the involvement of other CKI family members in mediating *Serpine1*-induced cell senescence cannot be ruled out.

DNA methylation regulates gene expression in various processes, including lung development [42]. Studies have shown that *Serpine1* expression is associated with DNA methylation in low-grade gliomas and adiposity [43, 44]. Here, we observed a decreased methylation of the *Serpine1* promoter in the lung tissues of neonatal mice with IUI, and inhibition of DNA methylation induced the upregulation of *Serpine1* expression. These results provide experimental evidence that *Serpine1* expression is regulated by DNA methylation. This is also the first study to report that *Serpine1* methylation is related to lung development. Besides, we observed a higher Tet1 expression in neonatal mice with IUI than in control mice. We also observed a TNF-α-mediated increased expression of Tet1, leading to Serpine1 upregulation. Meanwhile, overexpression of Tet1 increased the transcriptional efficiency of methylated *Serpine1* promoter. These data suggest that IUI causes a TNF-α-mediated demethylation of the *Serpine1* promoter through elevation of Tet1.

For the first time, we identified the TFs that regulated the expression of *Serpine1*, including Rela, Rest, and Hifl1a. Moreover, transcriptional activity of Rela, Rest, and Hifl1a on the *Serpine1* promoter was repressed by DNA methylation. DNA pull-down and ChIP assays revealed that the *Serpine1* promoter was co-occupied by Rela and Hdac2. DNA demethylation increased the binding of Rela to the *Serpine1* promoter and induced the release of Hdac2. It was noted that Hdac2 could repress *Serpine1* expression. HDAC modulators have been used in various therapeutic applications, such as for cancer and chronic inflammatory diseases. For example, theophylline, a bronchodilator that acts as an HDAC agonist, has been used to increase the sensitivity of steroids in patients with chronic obstructive pulmonary disease [45]. Therefore, it is possible to use Hdac2 activators alone or in combination to treat BPD.

## Conclusions

Collectively, the results elucidate that hypomethylation of the *Serpine1* promoter is associated with *Serpine1* upregulation and the progression of IUI-induced lung injury. IUI decreased the methylation level of the *Serpine1* promoter region by upregulating Tet1 expression via TNF-α, causing the release of Hdac2, and increased Rela binding to the *Serpine1* promoter, increasing *Serpine1* expression (Additional file 7: Fig. S7). Of course, there are some limitations for this study. First, the effect of TNF-α on *Serpine1* promoter methylation in the offspring require in vivo investigation using TNF-α treated pregnant mice*.* Second, the expression of Serpine1 should be further investigated in BPD patients. Nonetheless, the results of this study provide the primary evidence that altered *Serpine1* expression, mediated by DNA methylation, contributes to the pathogenesis of lung injury in neonatal mice with IUI. These findings suggest that therapeutic strategies targeting *Serpine1* methylation may effectively prevent IUI-induced lung injury.

## Methods

### Mouse model of IUI and *Serpine1* siRNA treatment

Pregnant C57BL/6 mice at embryonic day 12.5 (E12.5) were randomly divided into either control or LPS groups. Mice in the LPS group were intraperitoneally injected with 45 μg/kg LPS (*Escherichia coli* serotype 055: B5, Sigma-Aldrich), while the control group received an equal volume of saline. Lung tissues were collected from neonatal mice on postnatal day 1 (P1), P7, and P21, and stored at −80 °C before analysis. For *Serpine1* small-interfering RNA (siRNA) treatment, P3 neonatal mice with IUI were administered *Serpine1* siRNA (5`-CCAACAAGAGCCAAUCACATT-3`) or negative control siRNA (RiboBio) via intranasal delivery at a dose of 0.25 nmol/g/day for 4 days. Lung tissues were then collected on P7.

### Cell culture and treatments

MLE-12, A549, BEAS-2B and human epithelial kidney 293 T (HEK293T) cell lines were purchased from ATCC. MLE-12, A549 and BEAS-2B cells were cultured in DMEM/F12 medium supplemented with 10% fetal bovine serum (FBS, Gibco). HEK293T cells were grown in DMEM supplemented with 10% FBS. Cells were treated with 5-Aza-CdR (0.125, 0.25, 0.5 μM; Sigma), TSA (10, 25, 50 nM; Sigma), or TNF-α (10 ng/mL, 100 ng/mL; Abcam) for 24 h.

### Plasmid construction and transfection

*Serpine1* open-reading frame was amplified with the cDNA from C57BL/6 murine lung tissue and cloned into the pCI-neo vector (Promega). The JASPAR database was used to predict the potential transcription factor binding sites on the *Serpine1* promoter. *Rela, Hif1a,* and *Rest* were cloned into the pCMV-SPORT6 vector. Three shRNAs were designed to repress Hdac2 expression. All constructs were confirmed via sequencing. All vectors were transfected into cells using Lipofectamine 2000 (Invitrogen). Cells transfected with empty vector or scrambled shRNA vector were used as controls.

### Immunohistochemistry and immunofluorescence

Paraffin slices were dewaxed using xylene I and II (10 min each time), hydrated with gradient alcohol (100, 95, and 75%, 5 min each), and washed with PBS (three times, 5 min each). Antigen retrieval was performed in 1 × Tris–EDTA buffer at 100 ℃ for 20 min. After cooling to room temperature, the slices were incubated with 3% hydrogen peroxide solution for 10 min at room temperature and washed with PBS. Then, the slices were blocked with 3% BSA (Yeasen) for 60 min at 37 ℃ and probed with the following the primary antibodies: rabbit anti-CD31 (1:2000; Abcam), rabbit anti-SFTPC (1:2000; Abcam), rabbit anti-Tet1 (1:500; Abcam), and rabbit anti-Hdac2 (1:500; Abcam), in the wet box overnight at 4 ℃. For immunohistochemistry, the slices were washed with PBS (three times, 5 min each) and incubated with the HRP-linked goat anti-rabbit secondary antibody (1:500; Yeasen) for 60 min at 37 ℃. After PBS washes, the slices were stained with diaminobenzidine (DAB), counterstained with hematoxylin, dehydrated, cleared, sealed, observed under an inverted microscope, and photographed. For immunofluorescence, the slices were probed with Alexa Fluor 488-labeled donkey anti-rabbit IgG (1:1000; Invitrogen) for 60 min at 37 ℃. After three PBS washes (5 min each), the cells were stained with 4',6-diamidino-2-phenylindole (DAPI, Beyotime) for 10 min to visualize nuclei. Five fields under the microscope (× 200 or × 400) were randomly selected for evaluation using the ImageJ software.

### RT-qPCR

The total RNA was extracted using the RNeasy Mini Kit (Qiagen). The OD 260/280 nm ratio was measured using the NanoDrop 2000 spectrophotometer (Thermo Fisher Scientific). Reverse transcription was performed using TaKaRa PrimeScript 1st strand cDNA Synthesis kit (TaKaRa). Messenger RNA levels were quantified with a QuantStudio (TM) 7 Flex System (Applied Biosystems) using SYBR^®^ Green PCR Master Mix (AG BIO). The data were analysed using the 2^−ΔΔCt^ method.

### Western blot

Tissues or cells were lysed on ice with precooled lysis buffer for 30 min and centrifuged at 12 000 rpm for 10 min at 4 ℃. The supernatant was transferred to centrifuge tubes, and 5 × SDS loading buffer was added. Samples were denatured for 10 min at 99 ℃ and subjected to sodium dodecyl sulfate‐polyacrylamide gel electrophoresis and transferred onto a polyvinylidene fluoride membrane (Merck). The membrane was blocked with 5% BSA for 60 min at room temperature and incubated with the primary antibodies against Serpine1 (1:1000; Abcam), Nr4a1 (1:1000, Abcam), β-actin (1:1500, Yeasen), Gapdh (1:1500, Yeasen), and transferrin (1:1000, Beyotime) overnight at 4 ℃. After washing three times with 1 × TBST, the membrane was probed with the HRP-linked goat anti-rabbit secondary antibody (1:1000, Yeasen) for 60 min at room temperature and washed with 1 × TBST buffer. Proteins were visualized using electrogenerated chemiluminescence reagents (Epizyme) and detected with an ImageQuant LAS 4000 chemiluminescent Image Analyzer (General Electric). The relative densitometric density of the target band to the internal reference band was analysed using the ImageJ software.

### Cell proliferation assay

Cell proliferation staining was performed using a BeyoClick^™^ EdU Cell Proliferation Kit with Alexa Fluor 488 (Beyotime). Briefly, cells were transfected with the pCI-neo-Serpine1 or pCI-neo plasmid for 48 h. Subsequently, cells were incubated with EdU for 2 h at 37 ℃ and washed three times with PBS. Then, cells were fixed with 4% paraformaldehyde for 15 min at room temperature, washed three times with PBS and permeated with 0.3% Triton X-100 for another 15 min at room temperature. After three PBS washes, the cells were incubated with the Click Reaction Mixture for 30 min at room temperature with protection from light and then incubated with Hoechst 33,342 for 10 min. Five fields under the microscope (× 200) were randomly selected for the evaluation.

### Senescence-associated β-galactosidase activity

Cells were transfected with the pCI-neo-Serpine1 or pCI-neo plasmid for 48 h. The SA-β-Gal staining was performed using a Senescence β-galactosidase Staining kit (Beyotime), and the cells were examined under a light microscope (Olympus BX51). Cellular senescence was expressed as the percentage of SA-β-Gal-positive cells to the total cell number.

### Cell apoptosis analysis

Cells were transfected with the pCI-neo-Serpine1 or pCI-neo plasmid for 48 h. The apoptotic cells were detected using FITC Annexin V Apoptosis Detection Kit (Dojindo) followed by flow cytometry analysis.

### Cell cycle assay

Cells were transfected with the pCI-neo-Serpine1 or pCI-neo plasmid for 48 h. Then, cells were harvested and fixed overnight using 75% ethanol. After incubation with 50 μg/mL propidium iodide dye (Yeasen) and 100 μg/mL RNase A (Yeasen) for 30 min, flow cytometry (BD Biosciences) was performed to quantify the percentage of cells in each cell cycle phase (G0/G1, S, and G2/M).

### ROS content measurement

Cells were transfected with the pCI-neo-Serpine1 or pCI-neo plasmid for 24 h. Then, intracellular ROS content was measured using a ROS assay kit (Yeasen). The intracellular ROS content were expressed as fluorescence intensity. Excitation/emission wavelengths were 490/585 nm.

### Assessment of mitochondrial membrane potential

Cells were transfected with the pCI-neo-Serpine1 or pCI-neo plasmid for 24 h. Then, the membrane potential of isolated mitochondria was measured by JC-1 staining (Beyotime). Mitochondria containing JC-1 aggregates were detected in the red (excitation 525 nm, emission 590 nm) and those containing JC-1 monomers were detected in the green channels (excitation 490 nm, emission 530 nm). The ratio of red to green fluorescence signal intensity indicated the mitochondrial membrane potential.

### Cell immunofluorescence

Cells were fixed with 4% paraformaldehyde for 20 min at room temperature, washed three times with PBS (5 min each time), permeabilized with 0.25% Triton X-100 for 10 min at room temperature, washed with PBS (three times, 5 min each), and blocked with 5% BSA for 60 min at 37 ℃. The cells were then incubated with the following primary antibodies: rabbit anti-Tet1 (1:500, Abcam) and rabbit anti-H2AX (1:400, Abcam), overnight at 4 ℃. Cells were washed with PBS and probed with Alexa Fluor 488-labeled donkey anti-rabbit IgG (1:1000; Invitrogen) for 60 min at 37 ℃. After three PBS washes (5 min each), the nuclei were stained with DAPI for 10 min. Five microscope fields (× 200) were selected randomly for the evaluation. The mean fluorescence intensity was analysed using the ImageJ software.

### DNA methylation analysis

MethPrime program was used to predict the potential methylation sites of *Serpine1* and design the primers (Serpine1-BSP-F: 5′-GGAATTTGATATTTTTTAGTTTTTATATG-3′ and Serpine1-BSP-R: 5′-CCTACTTCTACCTCCTAAATACTCC-3′) for bisulfite sequencing PCR (BSP). Bisulfite conversion and purification were performed using the EpiTect bisulfite Kit (Qiagen) per the manufacturer’s instructions. Bisulfite-converted DNA was amplified with TaKaRa EpiTaq HS (TaKaRa), and the 139-bp PCR product was purified with the TaKaRa MiniBEST agarose Gel DNA Extraction Kit (TaKaRa). Amplified fragments were inserted into the pMD^™^18-T vector (TaKaRa) and transformed into *Escherichia coli* Trans5α. Plasmid DNA from individual clones was isolated and subjected to Sanger sequencing using M13F primers. The methylation status of each CpG locus was determined by sequence comparison using the QUMA software.

### Luciferase assay

The 2000 bp 5′UTR (untranslated region) fragment of *Serpine1* was amplified from genomic DNA of C57BL/6 mice using the primers Serpine1-NheI-F: 5'-AATGCTAGCCACCATGCAGATGTCTTCAGC-3' and Serpine1-EcoRI-R: 5'-ATTGAATTCTCAAGGCTCCATCACTTGGCCCAT-3'. The amplified PCR products were cloned into the PGL3-basic vector (Promega) to generate the PGL3-Serpine1 construct. The PGL3-Serpine1 vector was incubated with the CpG methyltransferase *M.SssI* (NEB) for 4 h at 37 °C to methylate all CpG residues, followed by purification of vectors with the Cycle Pure Kit (Omega). Complete methylation was verified via *BstUI* digestion (NEB). Vectors were co-transfected into 80% confluent MLE-12 or HEK293T cells. After 48 h of incubation, a Dual-Luciferase Reporter Assay System (Promega) was used to measure the relative luciferase activities.

### ChIP

ChIP was performed using a SimpleChIP Enzymatic chromatin IP Kit (Cell Signaling Technology) according to the manufacturer’s instructions. Treated or mock-treated (0.5 μM 5-Aza-CdR for 24 h) MLE-12 cells were crosslinked with 37% formaldehyde. Chromatin was fragmented via partial digestion with micrococcal nuclease before sonication. The lysate was incubated at 4 °C overnight with anti-Rela and anti-Hdac2 rabbit monoclonal antibodies, followed by incubation with Protein G Magnetic Beads for 2 h at 4 °C. After reversal of protein-DNA crosslinks at 65 °C for 2 h, the DNA was purified using DNA purification spin columns, and immunoprecipitated DNA was analysed via RT-qPCR using a pair of primers (Serpine1-Chip-F:5'-CTGCTGCCTCCCTTTATACCA-3' and Serpine1-Chip-R: 5'-CATGCCCTTTCACACGTACAC-3'). A distant region amplified by primers (NC-F: 5'-CCCCGTGACCTGGATTTGAT-3' and NC-R: 5'-TACTATTGTGGGGGTCGGGA-3') was used as control. Fold enrichment method was used to normalize ChIP-qPCR data.

### DNA pull-down

The *Serpine1* promoter was amplified from genomic DNA of C57BL/6 mice using a pair of 5'-biotinylated primers (Serpine1-biotin-F: 5'-TGGTATCTGTTTACTGGAAATGGAAAACA-3' and Serpine1-biotin-R: 5'-GCTCCGTTCCCTGGCTGA-3'), corresponding to -2000 bp upstream the translation initiation site of *Serpine1*. A distant region amplified by primers (NC-biotin-F: 5'- GGCACATACTCTGCCACTGA-3' and NC-biotin-R: 5'-TACTATTGTGGGGGTCGGGA-3') was used as the control. Nuclear protein from mouse lung tissues was extracted using NE-PER nuclear and cytoplasmic extraction reagents (Thermo Fisher Scientific). Next, 50 μg of Dynabeads^®^ M-280 Streptavidin (Invitrogen) was incubated with 1 μg of the promoter DNA for 1 h at 4 °C with rotation. After that, beads-DNA was washed three times with one volume of binding buffer (5 mM Tris–HCl, pH 7.5, 0.5 mM EDTA, and 1.0 M NaCl), and then resuspended in one volume of incubation buffer (50 mM Tris, 1 mM EDTA, 100 mM KCI, pH 7.0, 5% glycerol, and 0.1% Triton X-100). Nuclear proteins (100 μL) were added and incubated with beads overnight with rotating at 4 °C, followed by three washes before liquid chromatography-mass spectroscopy (LC-MS) and western blot analyses. The LC–MS was performed by Lumingbio Co., Ltd., (Shanghai, China).

### Statistical analysis

Data are presented as mean ± SEM and analysed using GraphPad Prism version 6.0 software. Statistical significance was determined using Student’s *t*-test or one-way ANOVA. *P* values < 0.05 were considered statistically significant. All the experiments were performed at least in three independent repeats.

## Supplementary Information

**Additional file 1: Figure S1. **Enrichment of differentially expressed genes in lung tissues of neonatal mice with intrauterine inflammation. **A **Immunofluorescence detection of Pro-SFTPC in the lung tissues of neonatal mice (green, DAPI: blue).** C**, **D **GO and KEGG enrichment of the upregulated genes.** E**, **F **GO and KEGG enrichment of the downregulated genes.*Q*-value ≤ 0.05 and fold-change ≥ 2 were used as the thresholds for screening the differentially expressed genes. Data show mean ± SEM; Data was analysed using unpaired *t*-tests.

**Additional file 2: Figure S2.** Genes expression levels in lung tissues of neonatal mice (**A**) The mRNA expression of differentially expressed genes analysed using RT-qPCR in lung tissues of neonatal mice. **B**, **C** Nr4a1 expression analysed using western blot in lung tissues of neonatal mice. **D** *Serpine1* expression in lung tissues of neonatal mice detected using RT-qPCR on P1, P7, and P21. **E**, **F** *Serpine1* expression analysed using RT-qPCR and western blot in siRNA-treated neonatal mouse lungs. Data show mean ± SEM; Data was analysed using unpaired *t*-tests.

**Additional file 3: Figure S3.** The effect of *Serpine1* overexpression in lung epithelial cells. **A** *Serpine1* expression analysed using RT-qPCR in *Serpine1*-overexpressing MLE-12 cells. **B–D Serpine1** expression analysed using RT-qPCR, western blot and Immunofluorescence in *Serpine1*-overexpressing A549 cells. **E**, **F **Apoptosis rate analysed using flow cytometry in Serpine1-overexpressing A549 cells. **G** p16 expressions analysed using western blot in *Serpine1*-overexpressing BEAS-2B cells. Data show mean ± SEM; Data was analysed using unpaired *t*-tests.

**Additional file 4: Figure S4.** Enrichment of differentially expressed genes in *Serpine1*-overexpressing MLE-12 cells. **A**, **B** GO and KEGG enrichment of the upregulated genes.** C**, **D** GO and KEGG enrichment of the downregulated genes. *Q*-value ≤ 0.05 and fold-change ≥ 2 were used as the thresholds for screening the differentially expressed genes.

**Additional file 5: Figure S5.** The expression of DNA methylation related enzymes in lung tissues and TNF-α-treated MLE-12 cells. **A** *Dnmt1*, *Dnmt3a*, *Mbd1,* and *Tet1* expression analysed using RT-qPCR in lung tissues of neonatal mice. **B** TNF-α expression analysed using RT-qPCR in lung tissues of neonatal mice. **C** *Dnmt1*, *Dnmt3a*, *Mbd1,* and *Tet1* expression analysed using RT-qPCR in TNF-α-treated MLE-12 cells. **D** *Serpine1* expression analysed using RT-qPCR in TNF-α-treated MLE-12 cells. Data show mean ± SEM; Data was analysed using unpaired *t*-tests.

**Additional file 6: Figure S6.** HDAC2 inhibition promoted the expression of *Serpine1*. **A** SDS-PAGE identification of the proteins pulled-down by DNA fragment of *Serpine1* promoter. The gel was stained with Coomassie blue. The indicated differential bands were excised with a scalpel and identified by LC-MS. **B **Total ion chromatogram of proteins. **C–E** *Serpine1* expression analysed using RT-qPCR and western blot in trichostatin A (TSA)-treated MLE-12 cells. Data show mean ± SEM; Data was analysed using unpaired *t*-tests and one-way ANOVA.

**Additional file 7: Figure S7.** Schematic representation of the molecular mechanisms underlying IUI-induced lung injury in neonatal mice.

## Data Availability

The datasets used and/or analysed during the current study are available from the corresponding author on reasonable request. GEO accession number: GSE212593, GSE212689.

## Abbreviations

IUI: Intrauterine inflammation
PTB: Preterm birth
BPD: Bronchopulmonary dysplasia
TNF-α: Tumor necrosis factor-α
IL: Interleukin
GO: Gene ontology
DEGs: Differentially expressed genes
KEGG: Kyoto encyclopedia of genes and genomes
CCND1: Cell cycle-related proteins cyclin D1
CCNE2: Cell cycle-related proteins cyclin E2
CDK2: Cyclin-dependent kinase 2
5-Aza-CdR: 5-Aza-2'-deoxycytidine
TF: Transcription factor
TSA: Trichostatin A
ROS: Reactive oxygen species
SIPS: Stress-induced premature senescence
RS: Replicative senescence
CKI: Cyclin-dependent kinase inhibitor
LPS: Lipopolysaccharide
MLE-12: Murine lung epithelial-12
DAPI: 4’,6-Diamidino-2-phenylindole
BSP: Bisulfite sequencing PCR

## Declarations

### Study approval

All animal procedures were approved by the institutional Laboratory Animal Welfare and Ethics Committee, approval number: GKLW (2019-76).
