# Supplementary figures and images for: Aberrant methylation of Serpine1 mediates lung injury in neonatal mice prenatally exposed to intrauterine inflammation
Source: Cell Biosci. 2022 Oct 1;12:164. doi: 10.1186/s13578-022-00901-8 (PMC9526974; doi:10.1186/s13578-022-00901-8)

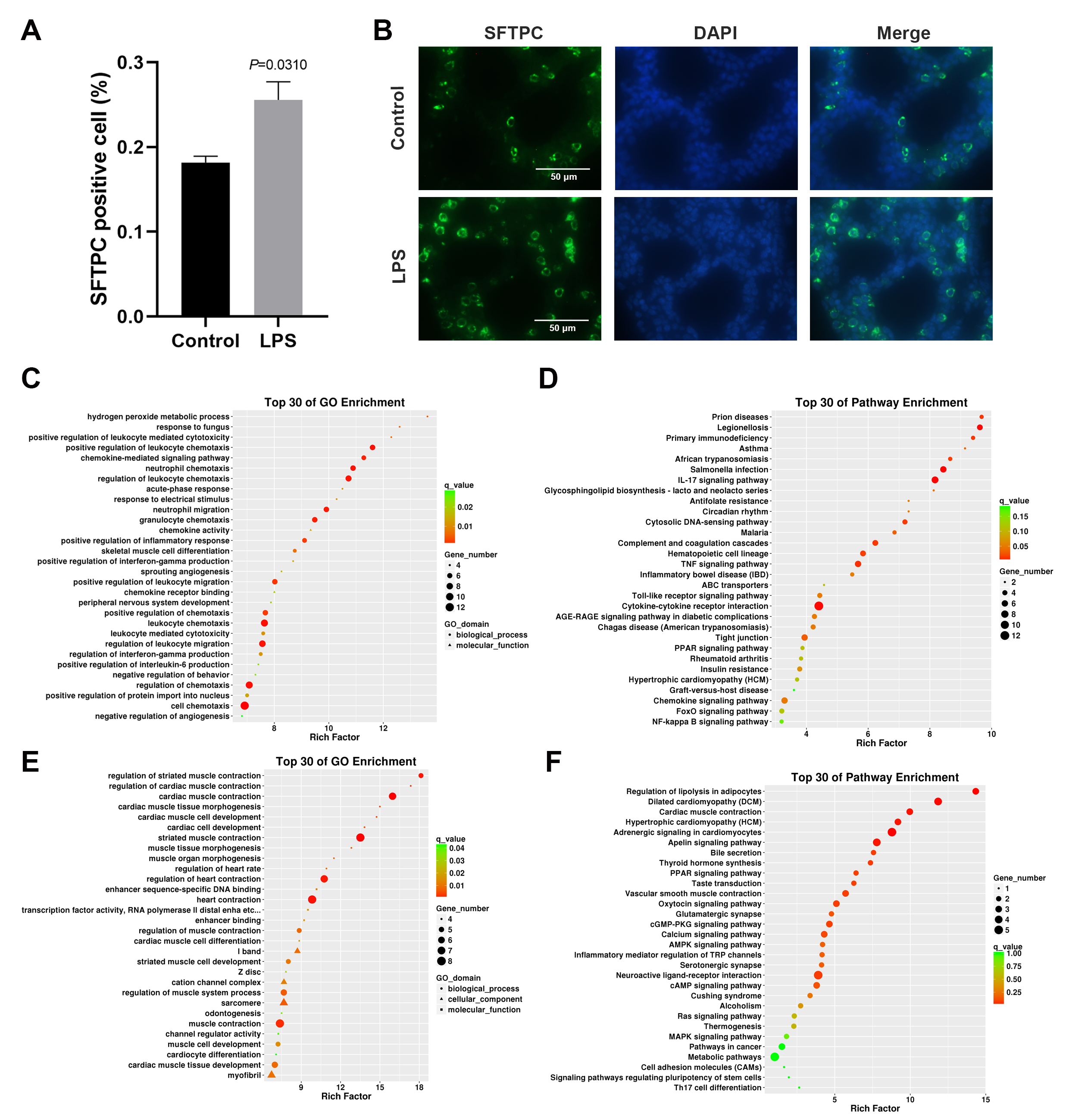

Supplement: Supplementary file 1 — Additional file 1: Figure S1. Enrichment of differentially expressed genes in lung tissues of neonatal mice with intrauterine inflammation. A Immunofluorescence detection of Pro-SFTPC in the lung tissues of neonatal mice (green, DAPI: blue). C, D GO and KEGG enrichment of the upregulated genes. E, F GO and KEGG enrichment of the downregulated genes.Q-value ≤ 0.05 and fold-change ≥ 2 were used as the thresholds for screening the differentially expressed genes. Data show mean ± SEM; Data was analysed using unpaired t-tests. [file 13578_2022_901_MOESM1_ESM.tif]

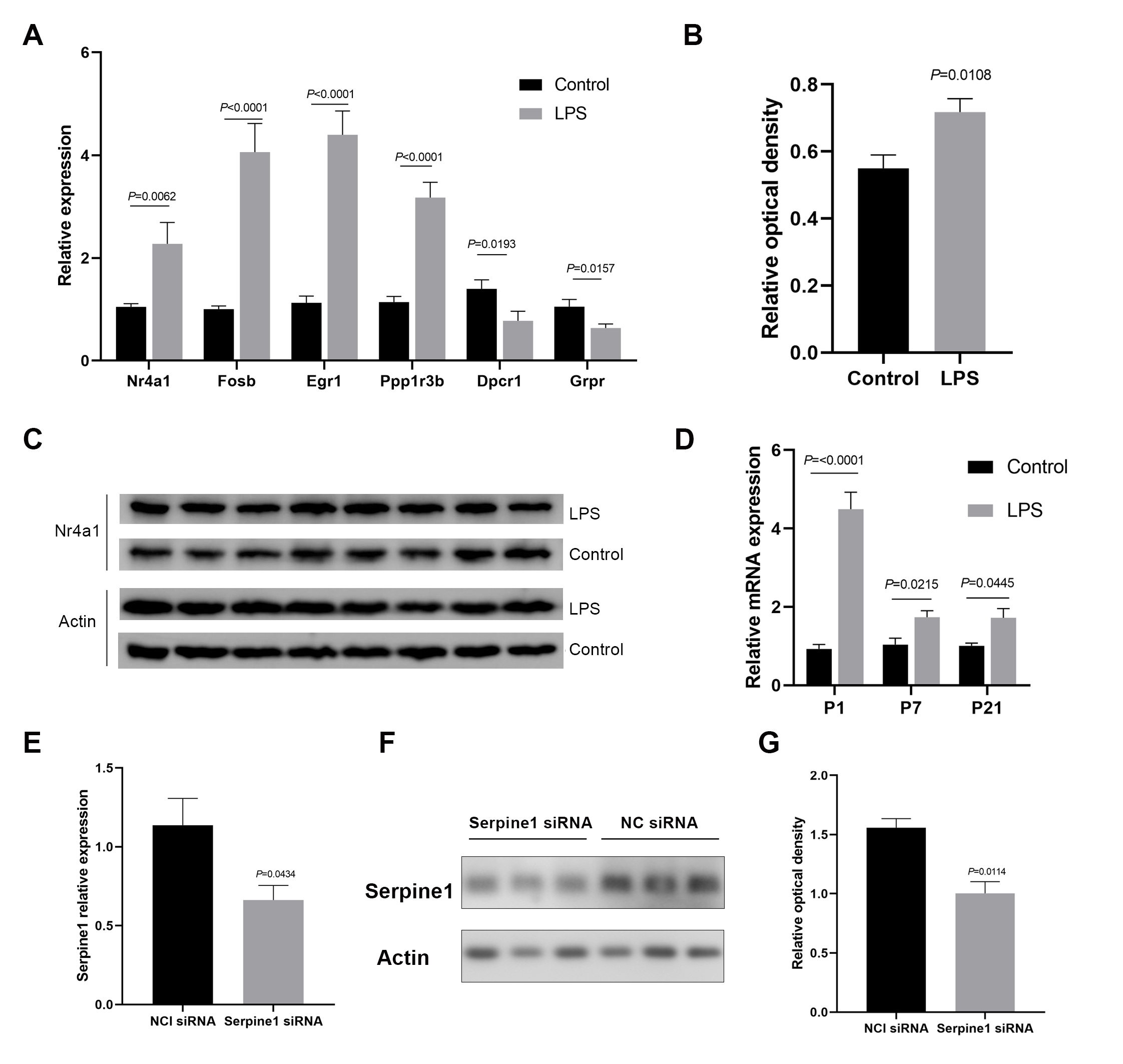

Supplement: Supplementary file 2 — Additional file 2: Figure S2. Genes expression levels in lung tissues of neonatal mice (A) The mRNA expression of differentially expressed genes analysed using RT-qPCR in lung tissues of neonatal mice. B, C Nr4a1 expression analysed using western blot in lung tissues of neonatal mice. D Serpine1 expression in lung tissues of neonatal mice detected using RT-qPCR on P1, P7, and P21. E, F Serpine1 expression analysed using RT-qPCR and western blot in siRNA-treated neonatal mouse lungs. Data show mean ± SEM; Data was analysed using unpaired t-tests. [file 13578_2022_901_MOESM2_ESM.tif]

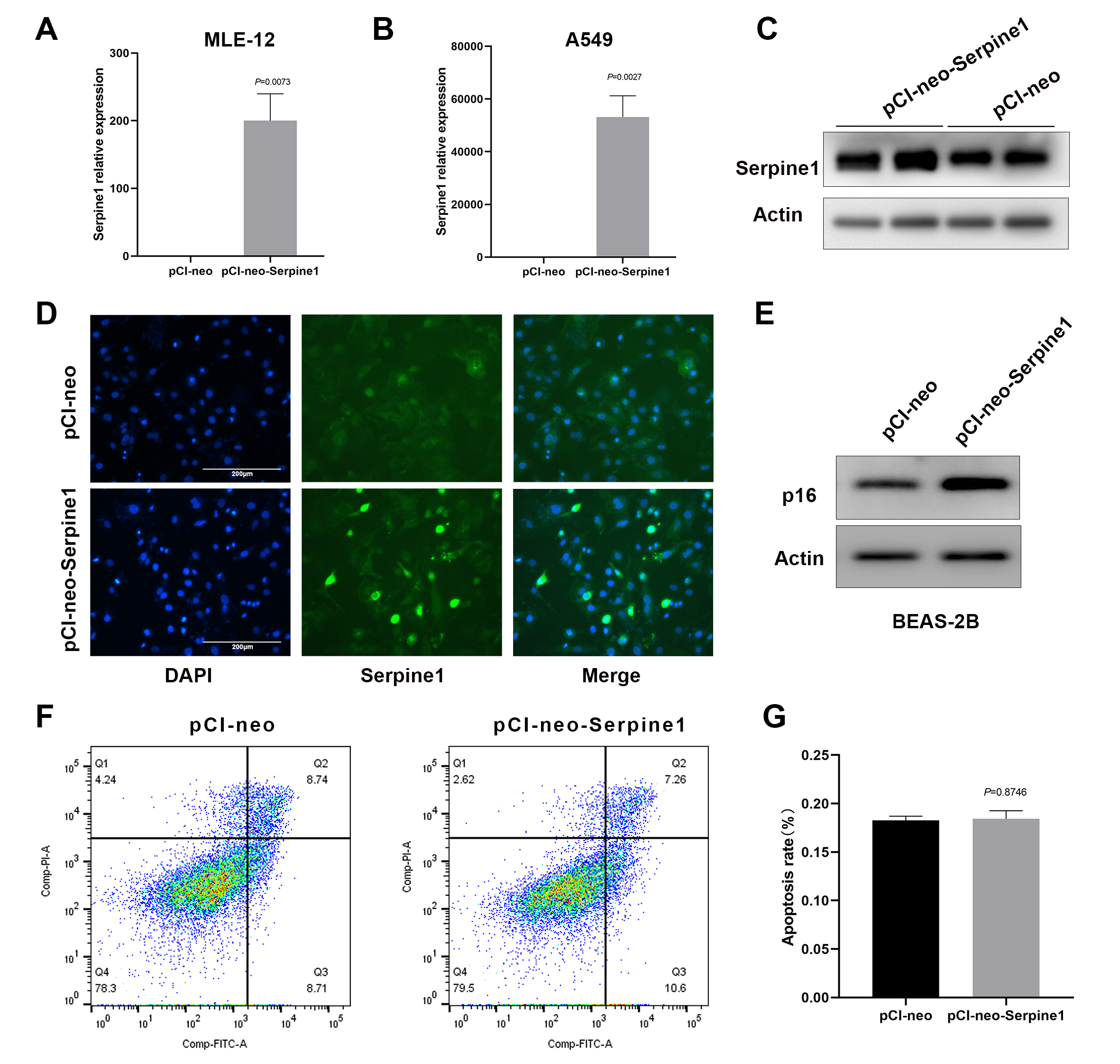

Supplement: Supplementary file 3 — Additional file 3: Figure S3. The effect of Serpine1 overexpression in lung epithelial cells. A Serpine1 expression analysed using RT-qPCR in Serpine1-overexpressing MLE-12 cells. B–D Serpine1 expression analysed using RT-qPCR, western blot and Immunofluorescence in Serpine1-overexpressing A549 cells. E, F Apoptosis rate analysed using flow cytometry in Serpine1-overexpressing A549 cells. G p16 expressions analysed using western blot in Serpine1-overexpressing BEAS-2B cells. Data show mean ± SEM; Data was analysed using unpaired t-tests. [file 13578_2022_901_MOESM3_ESM.tif]

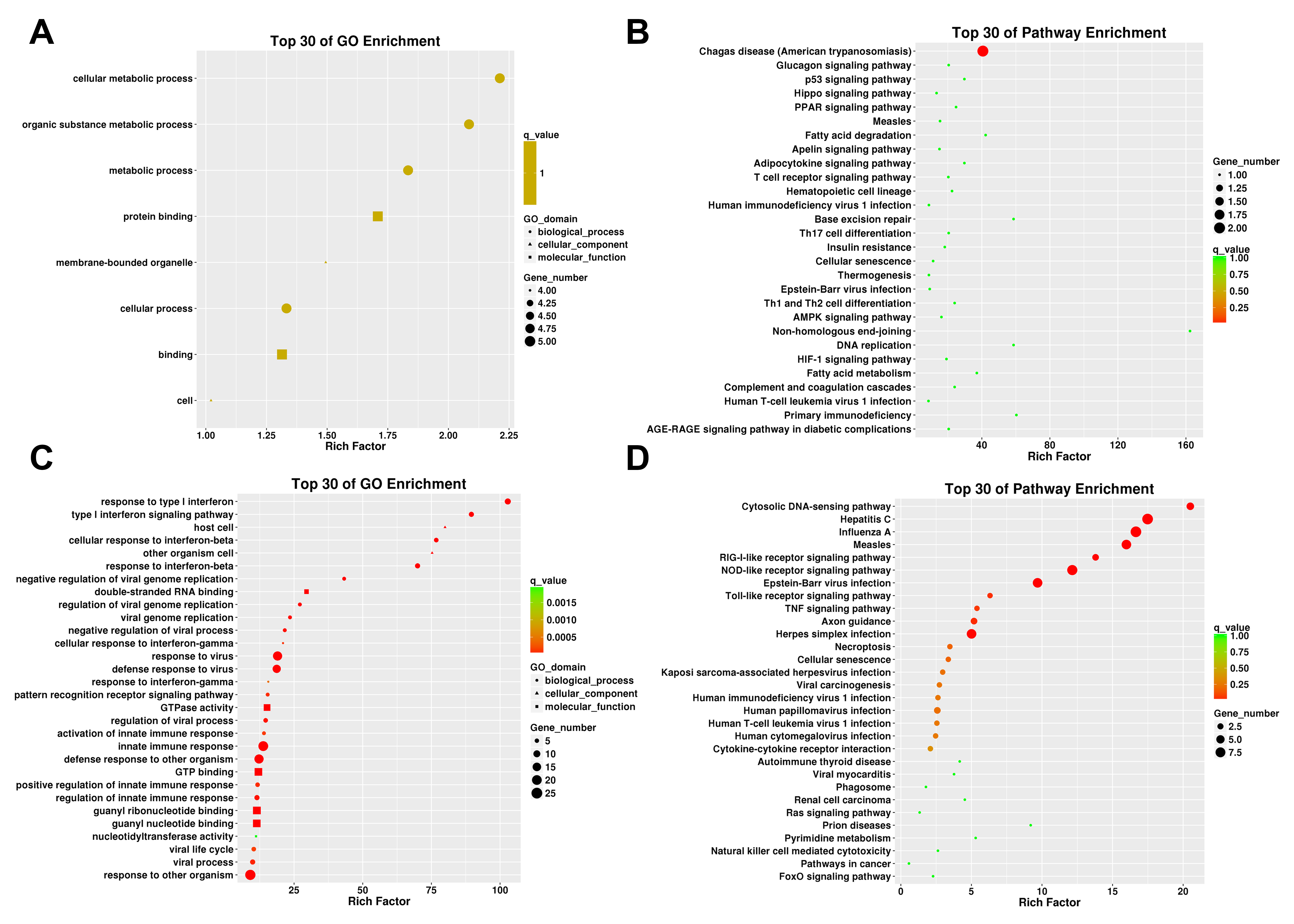

Supplement: Supplementary file 4 — Additional file 4: Figure S4. Enrichment of differentially expressed genes in Serpine1-overexpressing MLE-12 cells. A, B GO and KEGG enrichment of the upregulated genes. C, D GO and KEGG enrichment of the downregulated genes. Q-value ≤ 0.05 and fold-change ≥ 2 were used as the thresholds for screening the differentially expressed genes. [file 13578_2022_901_MOESM4_ESM.tif]

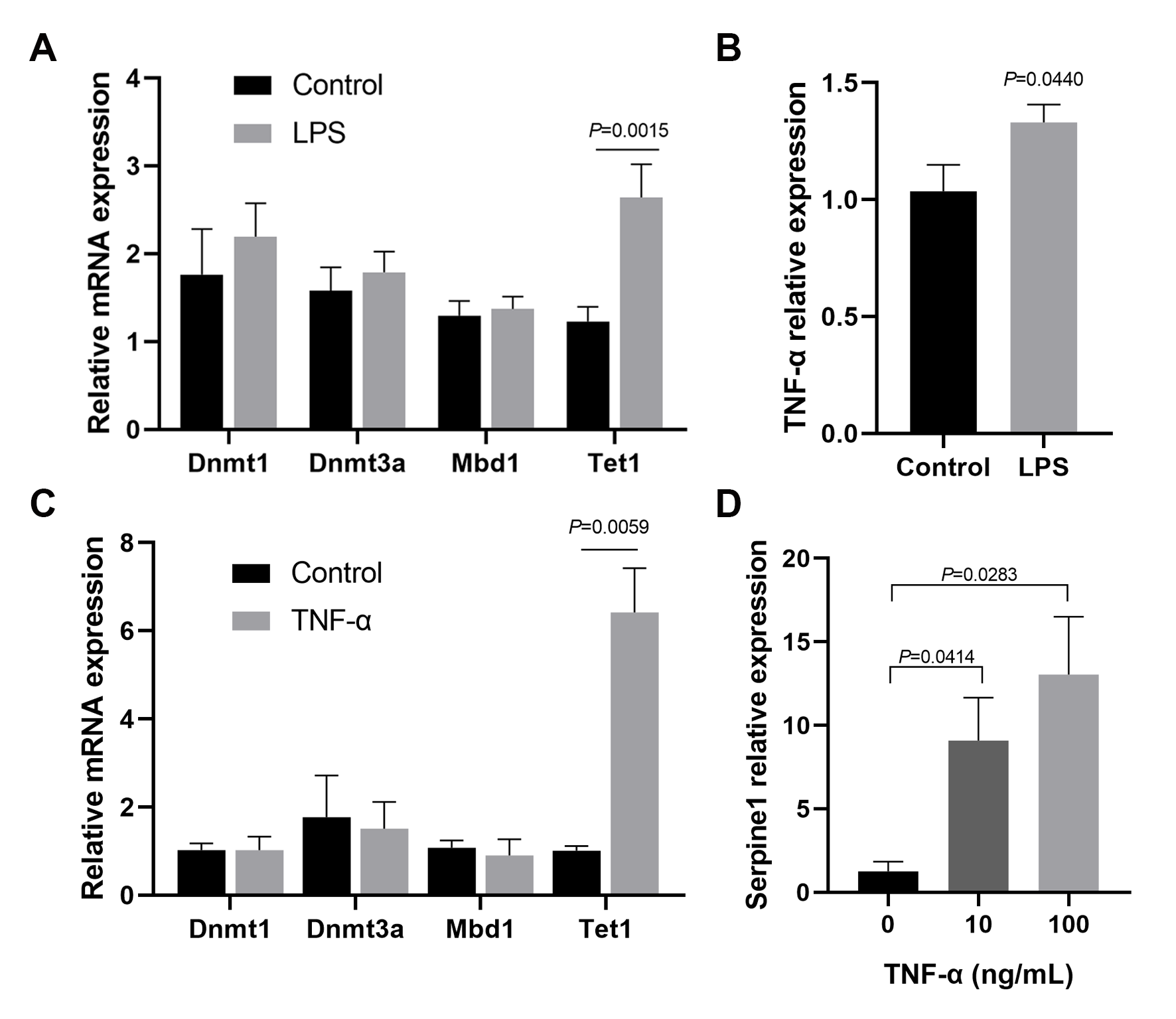

Supplement: Supplementary file 5 — Additional file 5: Figure S5. The expression of DNA methylation related enzymes in lung tissues and TNF-α-treated MLE-12 cells. A Dnmt1, Dnmt3a, Mbd1, and Tet1 expression analysed using RT-qPCR in lung tissues of neonatal mice. B TNF-α expression analysed using RT-qPCR in lung tissues of neonatal mice. C Dnmt1, Dnmt3a, Mbd1, and Tet1 expression analysed using RT-qPCR in TNF-α-treated MLE-12 cells. D Serpine1 expression analysed using RT-qPCR in TNF-α-treated MLE-12 cells. Data show mean ± SEM; Data was analysed using unpaired t-tests. [file 13578_2022_901_MOESM5_ESM.tif]

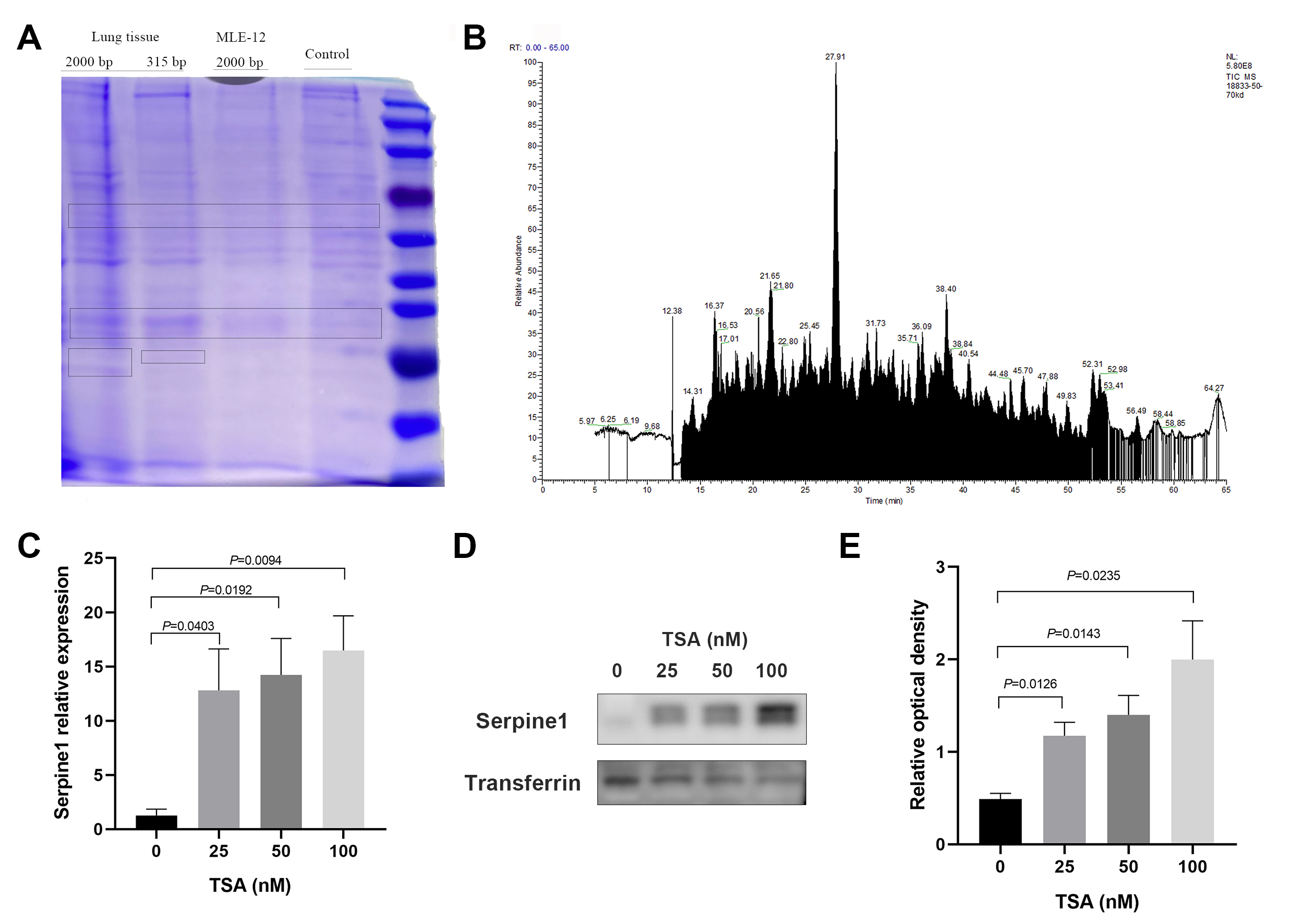

Supplement: Supplementary file 6 — Additional file 6: Figure S6. HDAC2 inhibition promoted the expression of Serpine1. A SDS-PAGE identification of the proteins pulled-down by DNA fragment of Serpine1 promoter. The gel was stained with Coomassie blue. The indicated differential bands were excised with a scalpel and identified by LC-MS. B Total ion chromatogram of proteins. C–E Serpine1 expression analysed using RT-qPCR and western blot in trichostatin A (TSA)-treated MLE-12 cells. Data show mean ± SEM; Data was analysed using unpaired t-tests and one-way ANOVA. [file 13578_2022_901_MOESM6_ESM.tif]

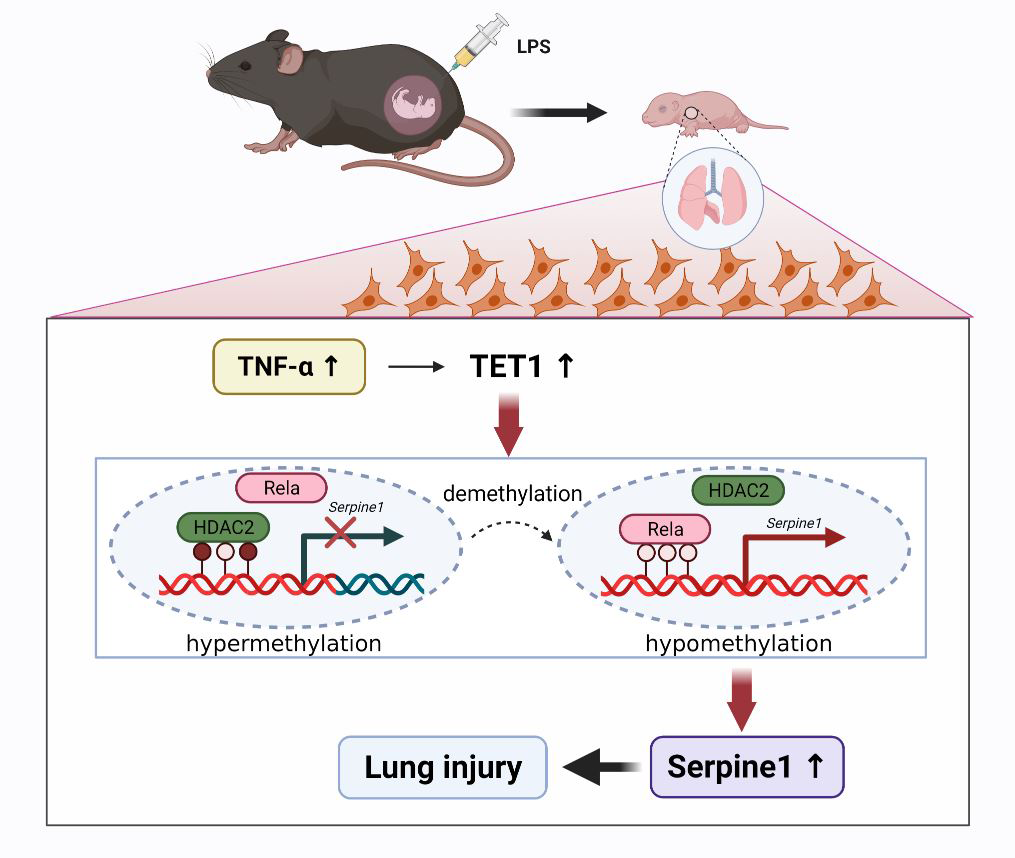

Supplement: Supplementary file 7 — Additional file 7: Figure S7. Schematic representation of the molecular mechanisms underlying IUI-induced lung injury in neonatal mice. [file 13578_2022_901_MOESM7_ESM.tif]
